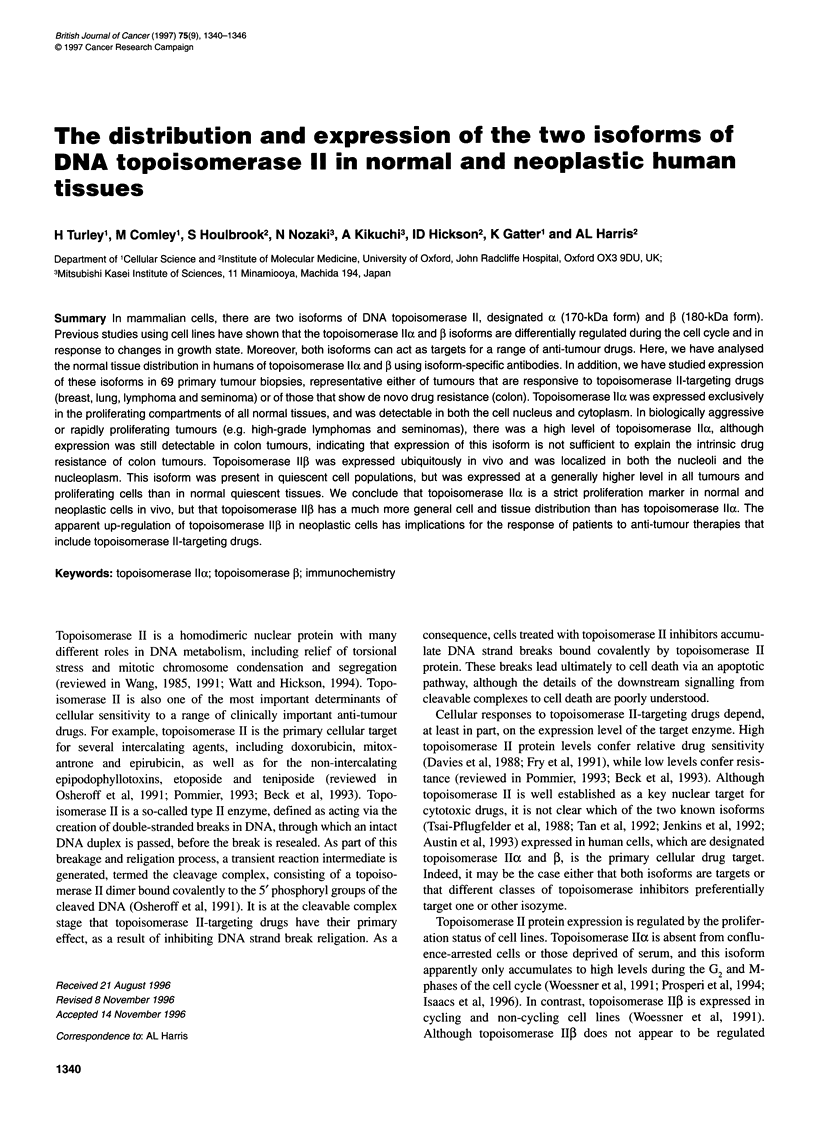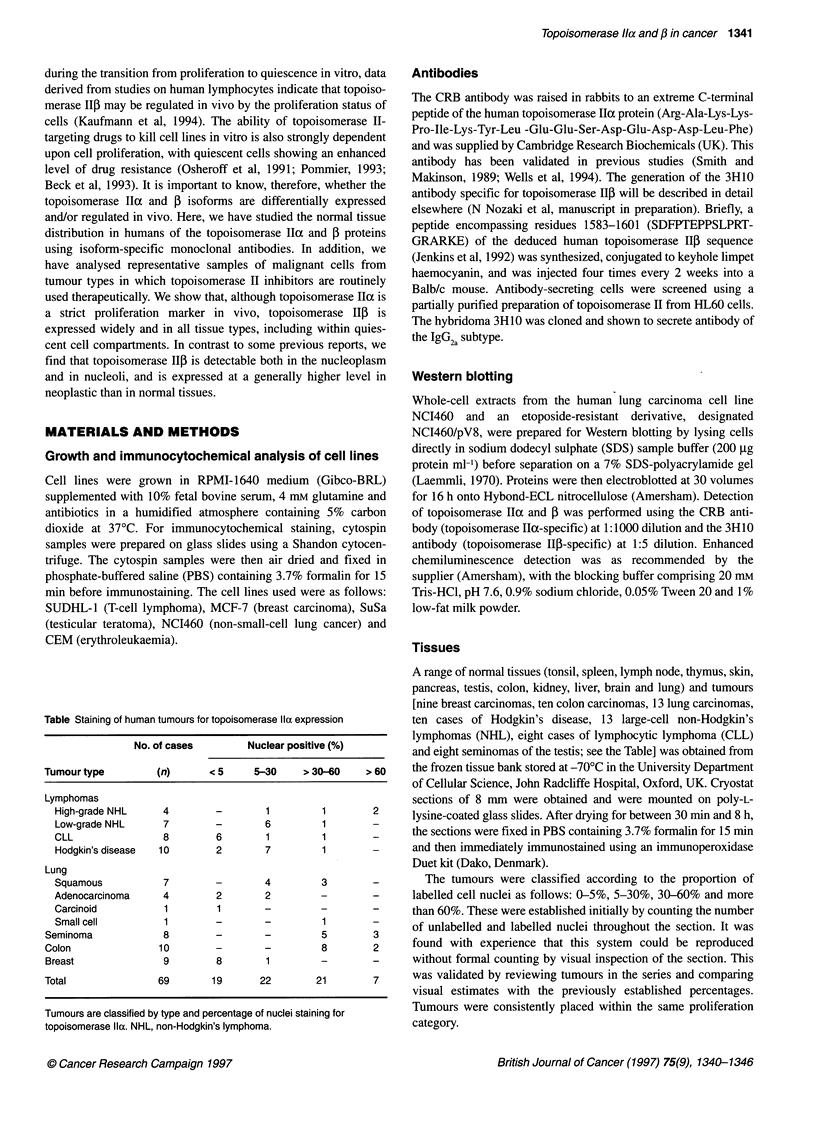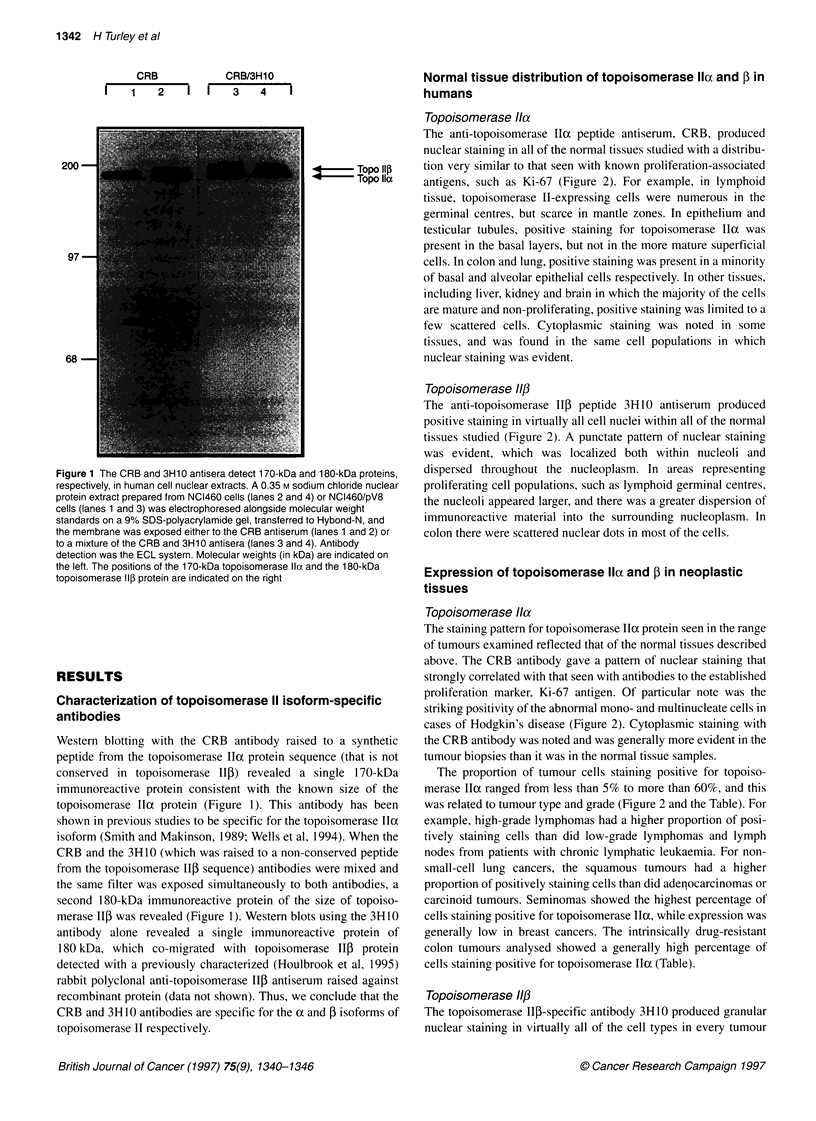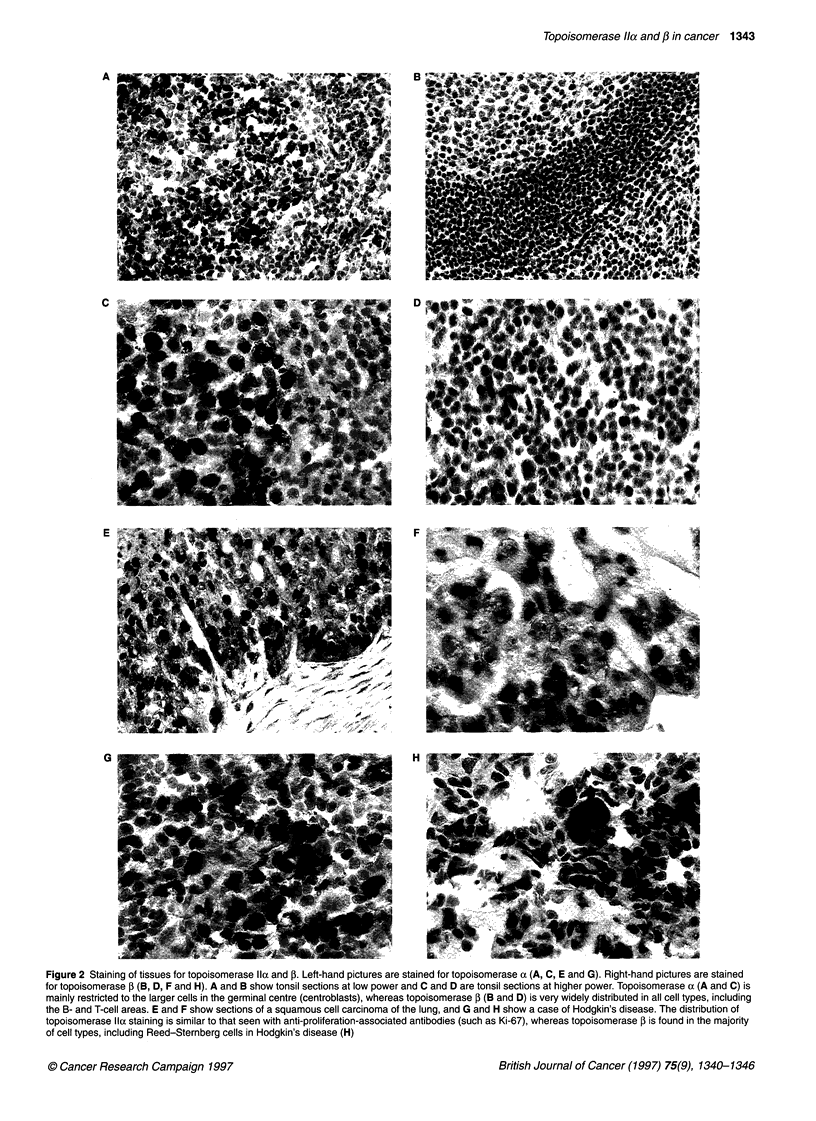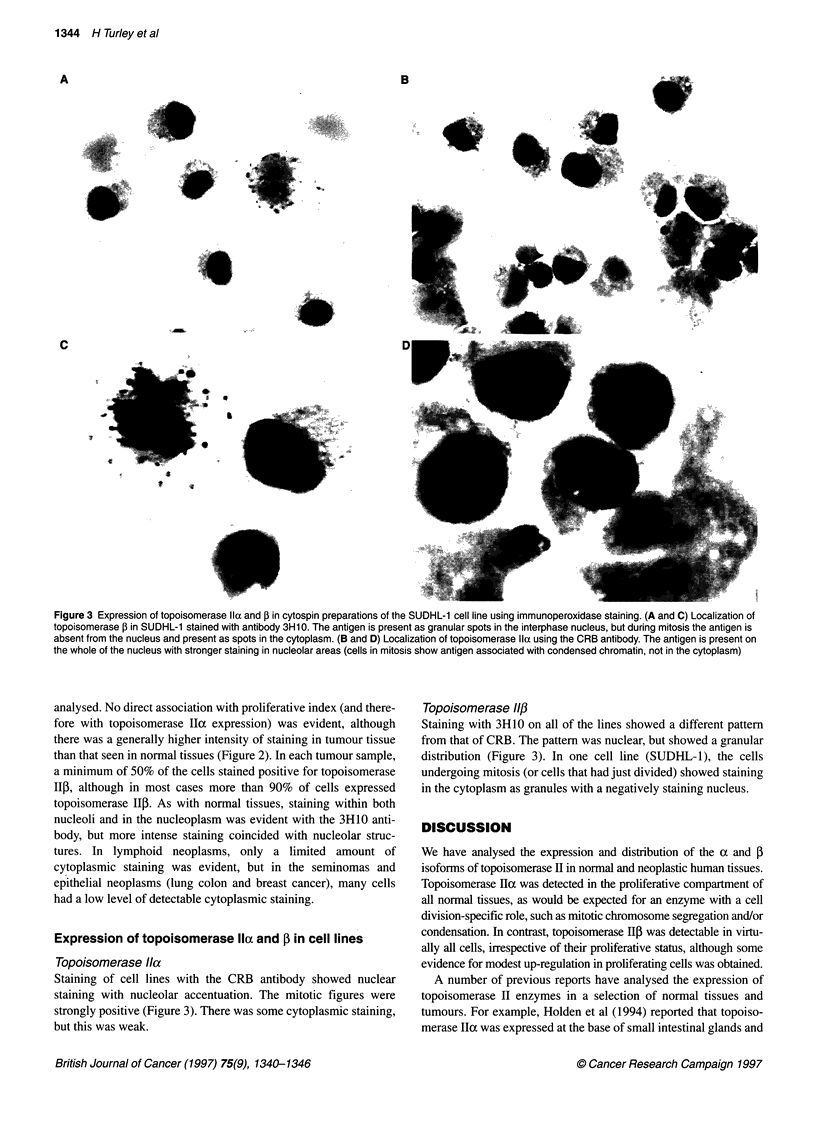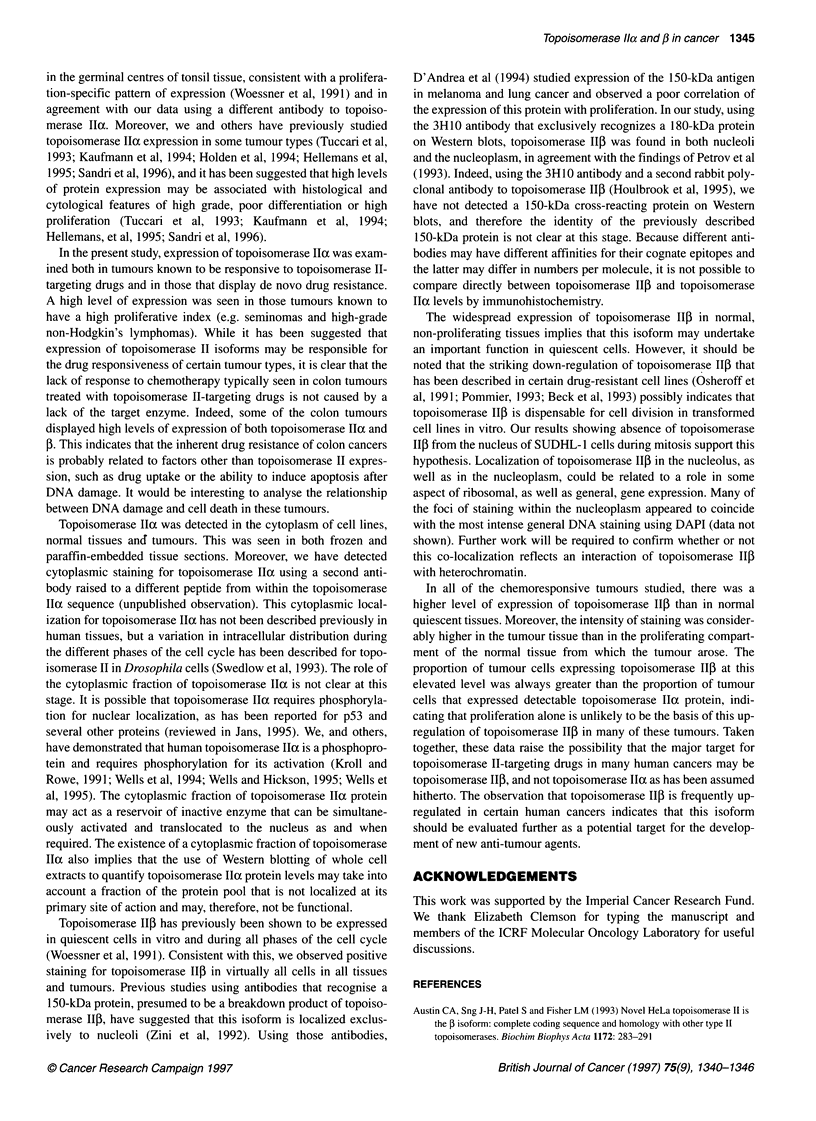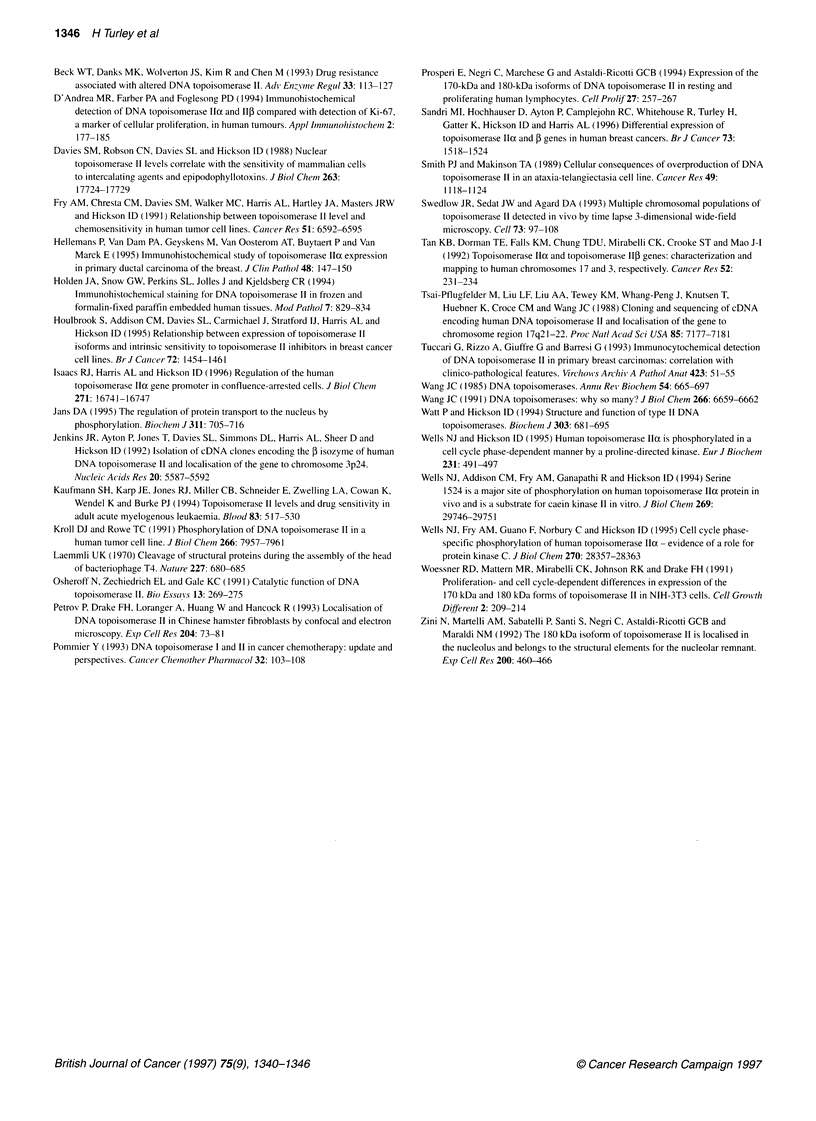# The distribution and expression of the two isoforms of DNA topoisomerase II in normal and neoplastic human tissues.

**DOI:** 10.1038/bjc.1997.227

**Published:** 1997

**Authors:** H. Turley, M. Comley, S. Houlbrook, N. Nozaki, A. Kikuchi, I. D. Hickson, K. Gatter, A. L. Harris

**Affiliations:** Department of Cellular Science, University of Oxford, John Radcliffe Hospital, UK.

## Abstract

**Images:**


*British Journal of Cancer* (1997) **75**(9), 1340–1346
© 1997 Cancer Research Campaign

### The distribution and expression of the two isoforms of DNA topoisomerase II in normal and neoplastic human tissues

**H Turley[1], M Comley[1], S Houlbrook[2], N Nozaki[3], A Kikuchi[3], ID Hickson[2], K Gatter[1] and AL Harris[2]**

Department of [1]Cellular Science and [2]Institute of Molecular Medicine, University of Oxford, John Radcliffe Hospital, Oxford OX3 9DU, UK;
[3]Mitsubishi Kasei Institute of Sciences, 11 Minamiooya, Machida 194, Japan

**Summary** In mammalian cells, there are two isoforms of DNA topoisomerase II, designated α (170-kDa form) and β (180-kDa form). Previous studies using cell lines have shown that the topoisomerase IIα and β isoforms are differentially regulated during the cell cycle and in response to changes in growth state. Moreover, both isoforms can act as targets for a range of anti-tumour drugs. Here, we have analysed the normal tissue distribution in humans of topoisomerase IIα and β using isoform-specific antibodies. In addition, we have studied expression of these isoforms in 69 primary tumour biopsies, representative either of tumours that are responsive to topoisomerase II-targeting drugs (breast, lung, lymphoma and seminoma) or of those that show de novo drug resistance (colon). Topoisomerase IIα was expressed exclusively in the proliferating compartments of all normal tissues, and was detectable in both the cell nucleus and cytoplasm. In biologically aggressive or rapidly proliferating tumours (e.g. high-grade lymphomas and seminomas), there was a high level of topoisomerase IIα, although expression was still detectable in colon tumours, indicating that expression of this isoform is not sufficient to explain the intrinsic drug resistance of colon tumours. Topoisomerase IIβ was expressed ubiquitously in vivo and was localized in both the nucleoli and the nucleoplasm. This isoform was present in quiescent cell populations, but was expressed at a generally higher level in all tumours and proliferating cells than in normal quiescent tissues. We conclude that topoisomerase IIα is a strict proliferation marker in normal and neoplastic cells in vivo, but that topoisomerase IIβ has a much more general cell and tissue distribution than has topoisomerase IIα. The apparent up-regulation of topoisomerase IIβ in neoplastic cells has implications for the response of patients to anti-tumour therapies that include topoisomerase II-targeting drugs.

**Keywords:** topoisomerase IIα; topoisomerase β; immunochemistry

Topoisomerase II is a homodimeric nuclear protein with many different roles in DNA metabolism, including relief of torsional stress and mitotic chromosome condensation and segregation (reviewed in Wang, 1985, 1991; Watt and Hickson, 1994). Topoisomerase II is also one of the most important determinants of cellular sensitivity to a range of clinically important anti-tumour drugs. For example, topoisomerase II is the primary cellular target for several intercalating agents, including doxorubicin, mitoxantrone and epirubicin, as well as for the non-intercalating epipodophyllotoxins, etoposide and teniposide (reviewed in Osheroff et al, 1991; Pommier, 1993; Beck et al, 1993). Topoisomerase II is a so-called type II enzyme, defined as acting via the creation of double-stranded breaks in DNA, through which an intact DNA duplex is passed, before the break is resealed. As part of this breakage and religation process, a transient reaction intermediate is generated, termed the cleavage complex, consisting of a topoisomerase II dimer bound covalently to the 5′ phosphoryl groups of the cleaved DNA (Osheroff et al, 1991). It is at the cleavable complex stage that topoisomerase II-targeting drugs have their primary effect, as a result of inhibiting DNA strand break religation. As a consequence, cells treated with topoisomerase II inhibitors accumulate DNA strand breaks bound covalently by topoisomerase II protein. These breaks lead ultimately to cell death via an apoptotic pathway, although the details of the downstream signalling from cleavable complexes to cell death are poorly understood.

*Received 21 August 1996*
*Revised 8 November 1996*
*Accepted 14 November 1996*

*Correspondence to*: AL Harris

Cellular responses to topoisomerase II-targeting drugs depend, at least in part, on the expression level of the target enzyme. High topoisomerase II protein levels confer relative drug sensitivity (Davies et al, 1988; Fry et al, 1991), while low levels confer resistance (reviewed in Pommier, 1993; Beck et al, 1993). Although topoisomerase II is well established as a key nuclear target for cytotoxic drugs, it is not clear which of the two known isoforms (Tsai-Pflugfelder et al, 1988; Tan et al, 1992; Jenkins et al, 1992; Austin et al, 1993) expressed in human cells, which are designated topoisomerase IIα and β, is the primary cellular drug target. Indeed, it may be the case either that both isoforms are targets or that different classes of topoisomerase inhibitors preferentially target one or other isozyme.

Topoisomerase II protein expression is regulated by the proliferation status of cell lines. Topoisomerase IIα is absent from confluence-arrested cells or those deprived of serum, and this isoform apparently only accumulates to high levels during the $G_2$ and M-phases of the cell cycle (Woessner et al, 1991; Prosperi et al, 1994; Isaacs et al, 1996). In contrast, topoisomerase IIβ is expressed in cycling and non-cycling cell lines (Woessner et al, 1991). Although topoisomerase IIβ does not appear to be regulated during the transition from proliferation to quiescence in vitro, data derived from studies on human lymphocytes indicate that topoisomerase IIβ may be regulated in vivo by the proliferation status of cells (Kaufmann et al, 1994). The ability of topoisomerase II-targeting drugs to kill cell lines in vitro is also strongly dependent upon cell proliferation, with quiescent cells showing an enhanced level of drug resistance (Osheroff et al, 1991; Pommier, 1993; Beck et al, 1993). It is important to know, therefore, whether the topoisomerase IIα and β isoforms are differentially expressed and/or regulated in vivo. Here, we have studied the normal tissue distribution in humans of the topoisomerase IIα and β proteins using isoform-specific monoclonal antibodies. In addition, we have analysed representative samples of malignant cells from tumour types in which topoisomerase II inhibitors are routinely used therapeutically. We show that, although topoisomerase IIα is a strict proliferation marker in vivo, topoisomerase IIβ is expressed widely and in all tissue types, including within quiescent cell compartments. In contrast to some previous reports, we find that topoisomerase IIβ is detectable both in the nucleoplasm and in nucleoli, and is expressed at a generally higher level in neoplastic than in normal tissues.

### MATERIALS AND METHODS

### Growth and immunocytochemical analysis of cell lines

Cell lines were grown in RPMI-1640 medium (Gibco-BRL) supplemented with 10% fetal bovine serum, 4 mM glutamine and antibiotics in a humidified atmosphere containing 5% carbon dioxide at 37°C. For immunocytochemical staining, cytospin samples were prepared on glass slides using a Shandon cytocentrifuge. The cytospin samples were then air dried and fixed in phosphate-buffered saline (PBS) containing 3.7% formalin for 15 min before immunostaining. The cell lines used were as follows: SUDHL-1 (T-cell lymphoma), MCF-7 (breast carcinoma), SuSa (testicular teratoma), NCI460 (non-small-cell lung cancer) and CEM (erythroleukaemia).

**Table** Staining of human tumours for topoisomerase IIα expression

| Tumour type | No. of cases (*n*) | Nuclear positive (%) < 5 | 5–30 | > 30–60 | > 60 |
|---|---|---|---|---|---|
| Lymphomas | | | | | |
| High-grade NHL | 4 | – | 1 | 1 | 2 |
| Low-grade NHL | 7 | – | 6 | 1 | – |
| CLL | 8 | 6 | 1 | 1 | – |
| Hodgkin's disease | 10 | 2 | 7 | 1 | – |
| Lung | | | | | |
| Squamous | 7 | – | 4 | 3 | – |
| Adenocarcinoma | 4 | 2 | 2 | – | – |
| Carcinoid | 1 | 1 | – | – | – |
| Small cell | 1 | – | – | 1 | – |
| Seminoma | 8 | – | – | 5 | 3 |
| Colon | 10 | – | – | 8 | 2 |
| Breast | 9 | 8 | 1 | – | – |
| Total | 69 | 19 | 22 | 21 | 7 |

Tumours are classified by type and percentage of nuclei staining for topoisomerase IIα. NHL, non-Hodgkin's lymphoma.

### Antibodies

The CRB antibody was raised in rabbits to an extreme C-terminal peptide of the human topoisomerase IIα protein (Arg-Ala-Lys-Lys-Pro-Ile-Lys-Tyr-Leu -Glu-Glu-Ser-Asp-Glu-Asp-Asp-Leu-Phe) and was supplied by Cambridge Research Biochemicals (UK). This antibody has been validated in previous studies (Smith and Makinson, 1989; Wells et al, 1994). The generation of the 3H10 antibody specific for topoisomerase IIβ will be described in detail elsewhere (N Nozaki et al, manuscript in preparation). Briefly, a peptide encompassing residues 1583–1601 (SDFPTEPPSLPRT-GRARKE) of the deduced human topoisomerase IIβ sequence (Jenkins et al, 1992) was synthesized, conjugated to keyhole limpet haemocyanin, and was injected four times every 2 weeks into a Balb/c mouse. Antibody-secreting cells were screened using a partially purified preparation of topoisomerase II from HL60 cells. The hybridoma 3H10 was cloned and shown to secrete antibody of the $IgG_{2a}$ subtype.

### Western blotting

Whole-cell extracts from the human lung carcinoma cell line NCI460 and an etoposide-resistant derivative, designated NCI460/pV8, were prepared for Western blotting by lysing cells directly in sodium dodecyl sulphate (SDS) sample buffer (200 μg protein $ml^{-1}$) before separation on a 7% SDS-polyacrylamide gel (Laemmli, 1970). Proteins were then electroblotted at 30 volumes for 16 h onto Hybond-ECL nitrocellulose (Amersham). Detection of topoisomerase IIα and β was performed using the CRB antibody (topoisomerase IIα-specific) at 1:1000 dilution and the 3H10 antibody (topoisomerase IIβ-specific) at 1:5 dilution. Enhanced chemiluminescence detection was as recommended by the supplier (Amersham), with the blocking buffer comprising 20 mM Tris-HCl, pH 7.6, 0.9% sodium chloride, 0.05% Tween 20 and 1% low-fat milk powder.

### Tissues

A range of normal tissues (tonsil, spleen, lymph node, thymus, skin, pancreas, testis, colon, kidney, liver, brain and lung) and tumours [nine breast carcinomas, ten colon carcinomas, 13 lung carcinomas, ten cases of Hodgkin's disease, 13 large-cell non-Hodgkin's lymphomas (NHL), eight cases of lymphocytic lymphoma (CLL) and eight seminomas of the testis; see the Table] was obtained from the frozen tissue bank stored at –70°C in the University Department of Cellular Science, John Radcliffe Hospital, Oxford, UK. Cryostat sections of 8 mm were obtained and were mounted on poly-L-lysine-coated glass slides. After drying for between 30 min and 8 h, the sections were fixed in PBS containing 3.7% formalin for 15 min and then immediately immunostained using an immunoperoxidase Duet kit (Dako, Denmark).

The tumours were classified according to the proportion of labelled cell nuclei as follows: 0–5%, 5–30%, 30–60% and more than 60%. These were established initially by counting the number of unlabelled and labelled nuclei throughout the section. It was found with experience that this system could be reproduced without formal counting by visual inspection of the section. This was validated by reviewing tumours in the series and comparing visual estimates with the previously established percentages. Tumours were consistently placed within the same proliferation category.

© Cancer Research Campaign 1997

**Figure 1** The CRB and 3H10 antisera detect 170-kDa and 180-kDa proteins, respectively, in human cell nuclear extracts. A 0.35 M sodium chloride nuclear protein extract prepared from NCI460 cells (lanes 2 and 4) or NCI460/pV8 cells (lanes 1 and 3) was electrophoresed alongside molecular weight standards on a 9% SDS-polyacrylamide gel, transferred to Hybond-N, and the membrane was exposed either to the CRB antiserum (lanes 1 and 2) or to a mixture of the CRB and 3H10 antisera (lanes 3 and 4). Antibody detection was the ECL system. Molecular weights (in kDa) are indicated on the left. The positions of the 170-kDa topoisomerase IIα and the 180-kDa topoisomerase IIβ protein are indicated on the right

### RESULTS

### Characterization of topoisomerase II isoform-specific antibodies

Western blotting with the CRB antibody raised to a synthetic peptide from the topoisomerase IIα protein sequence (that is not conserved in topoisomerase IIβ) revealed a single 170-kDa immunoreactive protein consistent with the known size of the topoisomerase IIα protein (Figure 1). This antibody has been shown in previous studies to be specific for the topoisomerase IIα isoform (Smith and Makinson, 1989; Wells et al, 1994). When the CRB and the 3H10 (which was raised to a non-conserved peptide from the topoisomerase IIβ sequence) antibodies were mixed and the same filter was exposed simultaneously to both antibodies, a second 180-kDa immunoreactive protein of the size of topoisomerase IIβ was revealed (Figure 1). Western blots using the 3H10 antibody alone revealed a single immunoreactive protein of 180 kDa, which co-migrated with topoisomerase IIβ protein detected with a previously characterized (Houlbrook et al, 1995) rabbit polyclonal anti-topoisomerase IIβ antiserum raised against recombinant protein (data not shown). Thus, we conclude that the CRB and 3H10 antibodies are specific for the α and β isoforms of topoisomerase II respectively.

### Normal tissue distribution of topoisomerase IIα and β in humans

### *Topoisomerase IIα*

The anti-topoisomerase IIα peptide antiserum, CRB, produced nuclear staining in all of the normal tissues studied with a distribution very similar to that seen with known proliferation-associated antigens, such as Ki-67 (Figure 2). For example, in lymphoid tissue, topoisomerase II-expressing cells were numerous in the germinal centres, but scarce in mantle zones. In epithelium and testicular tubules, positive staining for topoisomerase IIα was present in the basal layers, but not in the more mature superficial cells. In colon and lung, positive staining was present in a minority of basal and alveolar epithelial cells respectively. In other tissues, including liver, kidney and brain in which the majority of the cells are mature and non-proliferating, positive staining was limited to a few scattered cells. Cytoplasmic staining was noted in some tissues, and was found in the same cell populations in which nuclear staining was evident.

### *Topoisomerase IIβ*

The anti-topoisomerase IIβ peptide 3H10 antiserum produced positive staining in virtually all cell nuclei within all of the normal tissues studied (Figure 2). A punctate pattern of nuclear staining was evident, which was localized both within nucleoli and dispersed throughout the nucleoplasm. In areas representing proliferating cell populations, such as lymphoid germinal centres, the nucleoli appeared larger, and there was a greater dispersion of immunoreactive material into the surrounding nucleoplasm. In colon there were scattered nuclear dots in most of the cells.

### Expression of topoisomerase IIα and β in neoplastic tissues

### *Topoisomerase IIα*

The staining pattern for topoisomerase IIα protein seen in the range of tumours examined reflected that of the normal tissues described above. The CRB antibody gave a pattern of nuclear staining that strongly correlated with that seen with antibodies to the established proliferation marker, Ki-67 antigen. Of particular note was the striking positivity of the abnormal mono- and multinucleate cells in cases of Hodgkin's disease (Figure 2). Cytoplasmic staining with the CRB antibody was noted and was generally more evident in the tumour biopsies than it was in the normal tissue samples.

The proportion of tumour cells staining positive for topoisomerase IIα ranged from less than 5% to more than 60%, and this was related to tumour type and grade (Figure 2 and the Table). For example, high-grade lymphomas had a higher proportion of positively staining cells than did low-grade lymphomas and lymph nodes from patients with chronic lymphatic leukaemia. For non-small-cell lung cancers, the squamous tumours had a higher proportion of positively staining cells than did adenocarcinomas or carcinoid tumours. Seminomas showed the highest percentage of cells staining positive for topoisomerase IIα, while expression was generally low in breast cancers. The intrinsically drug-resistant colon tumours analysed showed a generally high percentage of cells staining positive for topoisomerase IIα (Table).

### *Topoisomerase IIβ*

The topoisomerase IIβ-specific antibody 3H10 produced granular nuclear staining in virtually all of the cell types in every tumour

© Cancer Research Campaign 1997

**Figure 2** Staining of tissues for topoisomerase IIα and β. Left-hand pictures are stained for topoisomerase α (**A, C, E** and **G**). Right-hand pictures are stained for topoisomerase β (**B, D, F** and **H**). **A** and **B** show tonsil sections at low power and **C** and **D** are tonsil sections at higher power. Topoisomerase α (**A** and **C**) is mainly restricted to the larger cells in the germinal centre (centroblasts), whereas topoisomerase β (**B** and **D**) is very widely distributed in all cell types, including the B- and T-cell areas. **E** and **F** show sections of a squamous cell carcinoma of the lung, and **G** and **H** show a case of Hodgkin's disease. The distribution of topoisomerase IIα staining is similar to that seen with anti-proliferation-associated antibodies (such as Ki-67), whereas topoisomerase β is found in the majority of cell types, including Reed–Sternberg cells in Hodgkin's disease (**H**)

© Cancer Research Campaign 1997

**Figure 3** Expression of topoisomerase IIα and β in cytospin preparations of the SUDHL-1 cell line using immunoperoxidase staining. (**A** and **C**) Localization of topoisomerase β in SUDHL-1 stained with antibody 3H10. The antigen is present as granular spots in the interphase nucleus, but during mitosis the antigen is absent from the nucleus and present as spots in the cytoplasm. (**B** and **D**) Localization of topoisomerase IIα using the CRB antibody. The antigen is present on the whole of the nucleus with stronger staining in nucleolar areas (cells in mitosis show antigen associated with condensed chromatin, not in the cytoplasm)

analysed. No direct association with proliferative index (and therefore with topoisomerase IIα expression) was evident, although there was a generally higher intensity of staining in tumour tissue than that seen in normal tissues (Figure 2). In each tumour sample, a minimum of 50% of the cells stained positive for topoisomerase IIβ, although in most cases more than 90% of cells expressed topoisomerase IIβ. As with normal tissues, staining within both nucleoli and in the nucleoplasm was evident with the 3H10 antibody, but more intense staining coincided with nucleolar structures. In lymphoid neoplasms, only a limited amount of cytoplasmic staining was evident, but in the seminomas and epithelial neoplasms (lung colon and breast cancer), many cells had a low level of detectable cytoplasmic staining.

### Expression of topoisomerase IIα and β in cell lines

*Topoisomerase IIα*

Staining of cell lines with the CRB antibody showed nuclear staining with nucleolar accentuation. The mitotic figures were strongly positive (Figure 3). There was some cytoplasmic staining, but this was weak.

*Topoisomerase IIβ*

Staining with 3H10 on all of the lines showed a different pattern from that of CRB. The pattern was nuclear, but showed a granular distribution (Figure 3). In one cell line (SUDHL-1), the cells undergoing mitosis (or cells that had just divided) showed staining in the cytoplasm as granules with a negatively staining nucleus.

### DISCUSSION

We have analysed the expression and distribution of the α and β isoforms of topoisomerase II in normal and neoplastic human tissues. Topoisomerase IIα was detected in the proliferative compartment of all normal tissues, as would be expected for an enzyme with a cell division-specific role, such as mitotic chromosome segregation and/or condensation. In contrast, topoisomerase IIβ was detectable in virtually all cells, irrespective of their proliferative status, although some evidence for modest up-regulation in proliferating cells was obtained.

A number of previous reports have analysed the expression of topoisomerase II enzymes in a selection of normal tissues and tumours. For example, Holden et al (1994) reported that topoisomerase IIα was expressed at the base of small intestinal glands and

© Cancer Research Campaign 1997

in the germinal centres of tonsil tissue, consistent with a proliferation-specific pattern of expression (Woessner et al, 1991) and in agreement with our data using a different antibody to topoisomerase IIα. Moreover, we and others have previously studied topoisomerase IIα expression in some tumour types (Tuccari et al, 1993; Kaufmann et al, 1994; Holden et al, 1994; Hellemans et al, 1995; Sandri et al, 1996), and it has been suggested that high levels of protein expression may be associated with histological and cytological features of high grade, poor differentiation or high proliferation (Tuccari et al, 1993; Kaufmann et al, 1994; Hellemans, et al, 1995; Sandri et al, 1996).

In the present study, expression of topoisomerase IIα was examined both in tumours known to be responsive to topoisomerase II-targeting drugs and in those that display de novo drug resistance. A high level of expression was seen in those tumours known to have a high proliferative index (e.g. seminomas and high-grade non-Hodgkin's lymphomas). While it has been suggested that expression of topoisomerase II isoforms may be responsible for the drug responsiveness of certain tumour types, it is clear that the lack of response to chemotherapy typically seen in colon tumours treated with topoisomerase II-targeting drugs is not caused by a lack of the target enzyme. Indeed, some of the colon tumours displayed high levels of expression of both topoisomerase IIα and β. This indicates that the inherent drug resistance of colon cancers is probably related to factors other than topoisomerase II expression, such as drug uptake or the ability to induce apoptosis after DNA damage. It would be interesting to analyse the relationship between DNA damage and cell death in these tumours.

Topoisomerase IIα was detected in the cytoplasm of cell lines, normal tissues and tumours. This was seen in both frozen and paraffin-embedded tissue sections. Moreover, we have detected cytoplasmic staining for topoisomerase IIα using a second antibody raised to a different peptide from within the topoisomerase IIα sequence (unpublished observation). This cytoplasmic localization for topoisomerase IIα has not been described previously in human tissues, but a variation in intracellular distribution during the different phases of the cell cycle has been described for topoisomerase II in *Drosophila* cells (Swedlow et al, 1993). The role of the cytoplasmic fraction of topoisomerase IIα is not clear at this stage. It is possible that topoisomerase IIα requires phosphorylation for nuclear localization, as has been reported for p53 and several other proteins (reviewed in Jans, 1995). We, and others, have demonstrated that human topoisomerase IIα is a phosphoprotein and requires phosphorylation for its activation (Kroll and Rowe, 1991; Wells et al, 1994; Wells and Hickson, 1995; Wells et al, 1995). The cytoplasmic fraction of topoisomerase IIα protein may act as a reservoir of inactive enzyme that can be simultaneously activated and translocated to the nucleus as and when required. The existence of a cytoplasmic fraction of topoisomerase IIα also implies that the use of Western blotting of whole cell extracts to quantify topoisomerase IIα protein levels may take into account a fraction of the protein pool that is not localized at its primary site of action and may, therefore, not be functional.

Topoisomerase IIβ has previously been shown to be expressed in quiescent cells in vitro and during all phases of the cell cycle (Woessner et al, 1991). Consistent with this, we observed positive staining for topoisomerase IIβ in virtually all cells in all tissues and tumours. Previous studies using antibodies that recognise a 150-kDa protein, presumed to be a breakdown product of topoisomerase IIβ, have suggested that this isoform is localized exclusively to nucleoli (Zini et al, 1992). Using those antibodies, D'Andrea et al (1994) studied expression of the 150-kDa antigen in melanoma and lung cancer and observed a poor correlation of the expression of this protein with proliferation. In our study, using the 3H10 antibody that exclusively recognizes a 180-kDa protein on Western blots, topoisomerase IIβ was found in both nucleoli and the nucleoplasm, in agreement with the findings of Petrov et al (1993). Indeed, using the 3H10 antibody and a second rabbit polyclonal antibody to topoisomerase IIβ (Houlbrook et al, 1995), we have not detected a 150-kDa cross-reacting protein on Western blots, and therefore the identity of the previously described 150-kDa protein is not clear at this stage. Because different antibodies may have different affinities for their cognate epitopes and the latter may differ in numbers per molecule, it is not possible to compare directly between topoisomerase IIβ and topoisomerase IIα levels by immunohistochemistry.

The widespread expression of topoisomerase IIβ in normal, non-proliferating tissues implies that this isoform may undertake an important function in quiescent cells. However, it should be noted that the striking down-regulation of topoisomerase IIβ that has been described in certain drug-resistant cell lines (Osheroff et al, 1991; Pommier, 1993; Beck et al, 1993) possibly indicates that topoisomerase IIβ is dispensable for cell division in transformed cell lines in vitro. Our results showing absence of topoisomerase IIβ from the nucleus of SUDHL-1 cells during mitosis support this hypothesis. Localization of topoisomerase IIβ in the nucleolus, as well as in the nucleoplasm, could be related to a role in some aspect of ribosomal, as well as general, gene expression. Many of the foci of staining within the nucleoplasm appeared to coincide with the most intense general DNA staining using DAPI (data not shown). Further work will be required to confirm whether or not this co-localization reflects an interaction of topoisomerase IIβ with heterochromatin.

In all of the chemoresponsive tumours studied, there was a higher level of expression of topoisomerase IIβ than in normal quiescent tissues. Moreover, the intensity of staining was considerably higher in the tumour tissue than in the proliferating compartment of the normal tissue from which the tumour arose. The proportion of tumour cells expressing topoisomerase IIβ at this elevated level was always greater than the proportion of tumour cells that expressed detectable topoisomerase IIα protein, indicating that proliferation alone is unlikely to be the basis of this up-regulation of topoisomerase IIβ in many of these tumours. Taken together, these data raise the possibility that the major target for topoisomerase II-targeting drugs in many human cancers may be topoisomerase IIβ, and not topoisomerase IIα as has been assumed hitherto. The observation that topoisomerase IIβ is frequently up-regulated in certain human cancers indicates that this isoform should be evaluated further as a potential target for the development of new anti-tumour agents.

### ACKNOWLEDGEMENTS

This work was supported by the Imperial Cancer Research Fund. We thank Elizabeth Clemson for typing the manuscript and members of the ICRF Molecular Oncology Laboratory for useful discussions.

### REFERENCES

Austin CA, Sng J-H, Patel S and Fisher LM (1993) Novel HeLa topoisomerase II is the β isoform: complete coding sequence and homology with other type II topoisomerases. *Biochim Biophys Acta* **1172**: 283–291

© Cancer Research Campaign 1997

Beck WT, Danks MK, Wolverton JS, Kim R and Chen M (1993) Drug resistance associated with altered DNA topoisomerase II. *Adv Enzyme Regul* **33**: 113–127

D'Andrea MR, Farber PA and Foglesong PD (1994) Immunohistochemical detection of DNA topoisomerase IIα and IIβ compared with detection of Ki-67, a marker of cellular proliferation, in human tumours. *Appl Immunohistochem* **2**: 177–185

Davies SM, Robson CN, Davies SL and Hickson ID (1988) Nuclear topoisomerase II levels correlate with the sensitivity of mammalian cells to intercalating agents and epipodophyllotoxins. *J Biol Chem* **263**: 17724–17729

Fry AM, Chresta CM, Davies SM, Walker MC, Harris AL, Hartley JA, Masters JRW and Hickson ID (1991) Relationship between topoisomerase II level and chemosensitivity in human tumor cell lines. *Cancer Res* **51**: 6592–6595

Hellemans P, Van Dam PA, Geyskens M, Van Oosterom AT, Buytaert P and Van Marck E (1995) Immunohistochemical study of topoisomerase IIα expression in primary ductal carcinoma of the breast. *J Clin Pathol* **48**: 147–150

Holden JA, Snow GW, Perkins SL, Jolles J and Kjeldsberg CR (1994) Immunohistochemical staining for DNA topoisomerase II in frozen and formalin-fixed paraffin embedded human tissues. *Mod Pathol* **7**: 829–834

Houlbrook S, Addison CM, Davies SL, Carmichael J, Stratford IJ, Harris AL and Hickson ID (1995) Relationship between expression of topoisomerase II isoforms and intrinsic sensitivity to topoisomerase II inhibitors in breast cancer cell lines. *Br J Cancer* **72**: 1454–1461

Isaacs RJ, Harris AL and Hickson ID (1996) Regulation of the human topoisomerase IIα gene promoter in confluence-arrested cells. *J Biol Chem* **271**: 16741–16747

Jans DA (1995) The regulation of protein transport to the nucleus by phosphorylation. *Biochem J* **311**: 705–716

Jenkins JR, Ayton P, Jones T, Davies SL, Simmons DL, Harris AL, Sheer D and Hickson ID (1992) Isolation of cDNA clones encoding the β isozyme of human DNA topoisomerase II and localisation of the gene to chromosome 3p24. *Nucleic Acids Res* **20**: 5587–5592

Kaufmann SH, Karp JE, Jones RJ, Miller CB, Schneider E, Zwelling LA, Cowan K, Wendel K and Burke PJ (1994) Topoisomerase II levels and drug sensitivity in adult acute myelogenous leukaemia. *Blood* **83**: 517–530

Kroll DJ and Rowe TC (1991) Phosphorylation of DNA topoisomerase II in a human tumor cell line. *J Biol Chem* **266**: 7957–7961

Laemmli UK (1970) Cleavage of structural proteins during the assembly of the head of bacteriophage T4. *Nature* **227**: 680–685

Osheroff N, Zechiedrich EL and Gale KC (1991) Catalytic function of DNA topoisomerase II. *Bio Essays* **13**: 269–275

Petrov P, Drake FH, Loranger A, Huang W and Hancock R (1993) Localisation of DNA topoisomerase II in Chinese hamster fibroblasts by confocal and electron microscopy. *Exp Cell Res* **204**: 73–81

Pommier Y (1993) DNA topoisomerase I and II in cancer chemotherapy: update and perspectives. *Cancer Chemother Pharmacol* **32**: 103–108

Prosperi E, Negri C, Marchese G and Astaldi-Ricotti GCB (1994) Expression of the 170-kDa and 180-kDa isoforms of DNA topoisomerase II in resting and proliferating human lymphocytes. *Cell Prolif* **27**: 257–267

Sandri MI, Hochhauser D, Ayton P, Camplejohn RC, Whitehouse R, Turley H, Gatter K, Hickson ID and Harris AL (1996) Differential expression of topoisomerase IIα and β genes in human breast cancers. *Br J Cancer* **73**: 1518–1524

Smith PJ and Makinson TA (1989) Cellular consequences of overproduction of DNA topoisomerase II in an ataxia-telangiectasia cell line. *Cancer Res* **49**: 1118–1124

Swedlow JR, Sedat JW and Agard DA (1993) Multiple chromosomal populations of topoisomerase II detected in vivo by time lapse 3-dimensional wide-field microscopy. *Cell* **73**: 97–108

Tan KB, Dorman TE, Falls KM, Chung TDU, Mirabelli CK, Crooke ST and Mao J-I (1992) Topoisomerase IIα and topoisomerase IIβ genes: characterization and mapping to human chromosomes 17 and 3, respectively. *Cancer Res* **52**: 231–234

Tsai-Pflugfelder M, Liu LF, Liu AA, Tewey KM, Whang-Peng J, Knutsen T, Huebner K, Croce CM and Wang JC (1988) Cloning and sequencing of cDNA encoding human DNA topoisomerase II and localisation of the gene to chromosome region 17q21–22. *Proc Natl Acad Sci USA* **85**: 7177–7181

Tuccari G, Rizzo A, Giuffre G and Barresi G (1993) Immunocytochemical detection of DNA topoisomerase II in primary breast carcinomas: correlation with clinico-pathological features. *Virchows Archiv A Pathol Anat* **423**: 51–55

Wang JC (1985) DNA topoisomerases. *Annu Rev Biochem* **54**: 665–697

Wang JC (1991) DNA topoisomerases: why so many? *J Biol Chem* **266**: 6659–6662

Watt P and Hickson ID (1994) Structure and function of type II DNA topoisomerases. *Biochem J* **303**: 681–695

Wells NJ and Hickson ID (1995) Human topoisomerase IIα is phosphorylated in a cell cycle phase-dependent manner by a proline-directed kinase. *Eur J Biochem* **231**: 491–497

Wells NJ, Addison CM, Fry AM, Ganapathi R and Hickson ID (1994) Serine 1524 is a major site of phosphorylation on human topoisomerase IIα protein in vivo and is a substrate for caein kinase II in vitro. *J Biol Chem* **269**: 29746–29751

Wells NJ, Fry AM, Guano F, Norbury C and Hickson ID (1995) Cell cycle phase-specific phosphorylation of human topoisomerase IIα – evidence of a role for protein kinase C. *J Biol Chem* **270**: 28357–28363

Woessner RD, Mattern MR, Mirabelli CK, Johnson RK and Drake FH (1991) Proliferation- and cell cycle-dependent differences in expression of the 170 kDa and 180 kDa forms of topoisomerase II in NIH-3T3 cells. *Cell Growth Different* **2**: 209–214

Zini N, Martelli AM, Sabatelli P, Santi S, Negri C, Astaldi-Ricotti GCB and Maraldi NM (1992) The 180 kDa isoform of topoisomerase II is localised in the nucleolus and belongs to the structural elements for the nucleolar remnant. *Exp Cell Res* **200**: 460–466

*© Cancer Research Campaign 1997*